# GPER1 and microRNA: Two Players in Breast Cancer Progression

**DOI:** 10.3390/ijms22010098

**Published:** 2020-12-24

**Authors:** Adele Vivacqua

**Affiliations:** Department of Pharmacy, Health and Nutrition Sciences, University of Calabria, 87036 Rende, Italy; adele.vivacqua@unical.it

**Keywords:** estrogens, breast cancer, CAFs, GPER1, miRNAs, microRNAs

## Abstract

Breast cancer is the main cause of morbidity and mortality in women worldwide. However, the molecular pathogenesis of breast cancer remains poorly defined due to its heterogeneity. Several studies have reported that G Protein-Coupled Estrogen Receptor 1 (GPER1) plays a crucial role in breast cancer progression, by binding to estrogens or synthetic agonists, like G-1, thus modulating genes involved in diverse biological events, such as cell proliferation, migration, apoptosis, and metastasis. In addition, it has been established that the dysregulation of short sequences of non-coding RNA, named microRNAs (miRNAs), is involved in various pathophysiological conditions, including breast cancer. Recent evidence has indicated that estrogens may regulate miRNA expression and therefore modulate the levels of their target genes, not only through the classical estrogen receptors (ERs), but also activating GPER1 signalling, hence suggesting an alternative molecular pathway involved in breast tumor progression. Here, the current knowledge about GPER1 and miRNA action in breast cancer is recapitulated, reporting recent evidence on the liaison of these two players in triggering breast tumorogenic effects. Elucidating the role of GPER1 and miRNAs in breast cancer might provide new tools for innovative approaches in anti-cancer therapy.

## 1. Introduction

Breast cancer is the most diagnosed cancer among women, with about two million cases each year and a mortality rate of 6.6% of all cancer deaths [1]. Breast cancer mortality in western countries has progressively decreased over the past 25 years and more than 90% of patients heal when the treatment starts at the early stages of the disease [1,2]. Therapy management of breast cancer depend on its histological and molecular characteristics, based primarily on the expression of estrogen receptors (ERs), progesterone receptor (PR) and ERBB2 receptor (HER2), which allow to distinct the breast carcinoma in diverse molecular subtypes (i.e., Luminal A and B, Her2-enriched, Basal- and Normal-like, ER-negative and Triple-Negative Breast Cancers, TNBC) [3]. Of note, four distinct subtypes of ER-negative breast cancer and six TNBC subtypes have been identified [3]. Based on this evidence, correct therapeutic approaches in breast cancer patients have become increasingly important to meliorate the response to conventional treatments. Despite advances in breast cancer therapeutic and diagnostic approaches, the management of metastasis derived from disseminated primary tumor cells remains an issue to address. [2,4]. It has been estimates that about 30% of women affected by breast cancer will develop metastasis in their lifetime [1]. Moreover, over 90% of the deaths of breast cancer patients are caused by metastasis [1]. Although the incidence of breast cancer rises with age, the rate of increase is slower after menopause, when estrogen production is reduced, thus suggesting the hormone-dependence of this neoplasia [4]. In this regard, it has been well established that the cumulative exposure of the breast tissue to estrogens may affect the cell division rate and cause increased cell proliferation, hence acting as an important risk factor in breast cancer development [4]. Estrogens, in particular 17β-estradiol (E2), exert important biological effects in a wide variety of normal and malignant tissues, through their nuclear receptors, ERα and ERβ [5]. Nevertheless, E2 also induce non-genomic effects binding to G protein coupled estrogen receptor 1 (GPER1) and activating rapid transduction pathways involved in neoplastic transformation and development [6]. In addition, it has been showed that E2 may regulate the expression of short sequences of non-coding RNA, named microRNAs (miRNAs) [7,8,9], whose dysregulation in diverse patho-physiological conditions, including breast cancer, it has been recently demonstrated [10,11].

Here, GPER1 and miRNAs involvement in breast cancer progression is reviewed, underlining the more recent action of GPER1 in modulating miRNA expression.

## 2. GPER1 History, Expression and Localization

Briefly, the history of GPER1 starts in the 1997 when a 7-transmembrane receptor belonging to the large family of membrane G-protein coupled receptor (GPCR), named GPR30, was identified and cloned [12]. Although GPR30 showed a similar structure to the interleukin 8 receptor and the angiotensin II receptor type 1 [13,14], the treatment with chemokines or peptides did not lead to the activation of this receptor [14], suggesting thus that GPR30 could be an orphan. Some years later, studies performed in breast cancer cells lacking ERs, but expressing GPR30, evidenced the ability of E2 to induce rapidly cell cascades and trigger growth effects through GPR30 [15,16]. Interestingly, these responses were blocked, silencing GPR30 expression [16]. Next, experimental data indicated a direct bind to GPR30 by E2, suggesting that it may work as a membrane-bound ER [17,18]. These findings, together with in vivo data showing that hepatic injury decreased by E2/GPER1/protein kinase A (PKA) pathway activation [19], allowed to name in 2007 GPR30 officially as the novel estrogen receptor GPER1 [20]. To date, in November 2020 “GPR30 or GPER1 or GPER and breast cancer” keywords in PubMed database yielded 1361 papers, whose about 89% published during the past decade [https://pubmed.ncbi.nlm.nih.gov/].

GPER1 gene, located on the chromosome 7p22.3, encodes a protein of a putative molecular mass of 41 kDa and high homology with its murine counterpart [21]. Similar to other GPCR, the N-terminal region of GPER1 is outside the cell, whereas the C-terminal region is located intracellularly [21].

Regarding GPER1 expression, its protein is not only expressed in normal and tumoral estrogen-responsive tissues [12,16,22,23], but also in several other tissues, such as brain, lung, liver, adipose tissue, vasculature, as well as in immune cells [24,25,26]. Interestingly, the expression of GPER1 is variable and may depend on the specie and tissue, as well as on the age and gender [27,28]. For instance, in the mammary gland, GPER1 expression is lower during puberty and then increases in sexual maturity stages [29]. In a number of cancer types, like breast, endometrial and ovarian tumors, high levels of GPER1 have been associated with larger tumor size, Her-2 expression and metastasis, as well as with poor survival [30,31,32,33]. However, it has also been reported that in breast cancer low levels of GPER are associated with metastatic lymph nodes, absence of ER expression, poor patient disease-free survival, and overall survival [34].

The significance of GPER1 expression related to ER-positive breast cancer and in TNBC has been investigated. GPER1 has been detected in about 50 to 60% of breast cancer tissues [35]. Among these, GPER1 expression has been evidenced in the majority of TNBC, whereas co-expression of GPER1 and ER was about 40% of all cases examined [35]. Notably, in patients treated with tamoxifen the expression of GPER1 was found increased and associated with a poor prognosis and relapse-free survival, suggesting that GPER1 expression in ER-positive breast cancer is correlated to tamoxifen resistance [36].

In TNBC, GPER1 expression is related with augmented recurrence and associated with a younger age and a more aggressive disease. After a 36-month follow-up, it has been reported that the rate survival in patients affected by TNBC and expressing low levels of GPER1 was 90%, whereas in the cohort with high GPER1 levels only 78% of the patients survived after this time period [34].

Moreover, GPER-1 in TNBC has been associated with a significant shorter overall survival and relapse-free survival of premenopausal patients [37].

GPER1 localization has been controversially discussed for many years. Similar to most trans-membrane GPCRs, GPER1 is integrated into the membrane and its ligand-activation lead to cAMP synthesis, a typical plasma membrane event [17,38,39,40]. Several studies confirm that GPER1 is mainly placed to the plasma membrane of diverse cells, like mammary and uterine epithelial cells [41,42], myometrium cells [43], renal epithelia cells [44,45,46], and hippocampal neurons [39,47]. However, the localization of GPER1 also seems to depend on species, tissue, and cell types. In this context, it has been reported an intracellular localization of GPER1, in particular bound to endoplasmic reticulum and co-localized in the Golgi apparatus and nuclear membrane in endothelial [48], vascular smooth muscle cells [48] and pancreatic islet cells [49,50] as well as in diverse cancer cell types [18]. Of note, an intranuclear localization of GPER1 it has also been shown in the more representative components of tumor stroma, especially of breast cancer, named cancer associated fibroblasts (CAFs) [51,52]. These data suggest that the cellular localization of GPER1 may be varied by specific environmental signalling.

## 3. GPER1 Ligands and Signalling

Although GPER is a very ubiquitous receptor in the cell, the endogenous and canonical ligand of GPER has not yet been found. Therefore, diverse computational and experimental studies have been performed in order to identify natural or synthetic ligands of GPER1 ([53,54] and reviewed therein). Apart from E2 that binds to GPER1 with an estimated binding affinity of 3–6 nM [17,18], a great number of other compounds have been identified, like genistein, quercetin, bisphenol A and many pesticides [53,55,56,57]. Interestingly, data have indicated that the modulators or antagonists of the classical ERs, such as tamoxifen, raloxifene, and fulvestrant, may act as GPER1 agonists [17,58]. In this context, discovery that tamoxifen is a GPER1-agonist led to the conclusion that GPER1 may contribute to the tamoxifen resistance in breast malignancy. Indeed, studies have indicated an increased expression of GPER1 in breast tumors with an acquired tamoxifen resistance [36].

Identification and synthesis in 2006 of the highly selective GPER1 agonist, G-1 [59] has allowed to analyse its specific signalling pathways and to obtained selective information on the GPER1 action in diverse cell contexts. Indeed, by experiments of binding, it was evidenced that G-1 has high affinity for GPER1 (Kd = 10 nM), but it binds to ERs at concentrations of 10µM [60]. The next year, two novel GPER1 specific agonists, named GPER1-L1 and GPER1-L2, with binding affinities of about 100 nM were synthesized [61]. Moreover, it was reported that propyl pyrazole triol (PPT), usually used as ERα specific agonist, may act as GPER1 agonist at concentrations as low as 10–100 nM, while the ERβ specific agonist diarylpropionitrile (DPN) had no effects on GPER1 activity [62].

As concern the antagonist compounds of GPER1, by screening a small molecules library (NIH-MLSMR), it was found G-15 as a selective GPER1 antagonist. G-15 is a synthetic substituted dihydroquinoline with similar structure as G-1, but lacking of the ethanone moiety [60]. G-15 shows a GPER binding affinity of 20 nM and only a minimal binding to ERs (Kd > 10 µM) [60]. Restoring the steric bulk of G-1, another GPER1 specific antagonist, named G-36, with an improved affinity to GPER1, was next developed [60]. Lappano et coll. reported that estriol, which is one of three endogenous estrogens (the other two are estradiol and estrone [63]), is an inhibitor of GPER1 activity [64]. As few years later, further experimental data showed that in breast cancer cells the synthetic molecule MIBE may bind to and block both ERα and GPER1 activity [61]. More recently, it has been identified the first peptide GPER1 ligand, termed ERalpha17p, corresponds to a portion of the hinge region/AF2 domain of the human ERα [65,66]. ERalpha17p shows an anti-tumoral activity in breast cancer cells [65,66].

A number of studies has indicated that, in estrogen-sensitive tumors, the activation of GPER1 induces several cascade responses leading to important biological events, like cell growth, migration and angiogenesis [62,67,68,69,70]. The network of signal transduction pathways mediated by GPER1 includes transactivation of the epidermal growth factor receptor (EGFR), activation of the mitogen activated protein kinase/extracellular regulated kinase (MAPK/ERK) and phosphatidylinositol 3-kinase (PI3K)/Akt cascades, calcium mobilization, and intracellular cyclic AMP production. In particular, in human breast cancer cells, the activation of MAPK/ERK signalling by E2-GPER1 binding involves the heterotrimeric G-proteins βγ-subunits and the cytosolic kinase src activation, suggesting the contribution of the heparin-bound EGF cleavage [15]. In particular, estrogenic GPER1 stimulation leads to the release of intracellular calcium from endoplasmic reticulum stores and the consequent activation of integrin α5β1, which in turn induces matrix metalloproteinase-dependent activation of the EGFR by release of membrane heparin-bound EGF [23]. In this manner, estrogenic action via GPER1 coordinates the release of local EGF-related polypeptides and EGFR phosphorylation, which, activating STAT5 and MAPK/ERK pathways, induces cellular activities associated with cell survival [23]. Besides ERK activation, the transactivation of the EGFR by E2 also activates PI3K/Akt pathway [70]. It has also been reported that, in breast cancer cells, the estrogenic stimulation of GPER1 causes activation of the Gαs protein which, in turn, leads to adenylyl cyclase activation and cAMP accumulation [17,21].

Growing evidence has shown that activation of GPER1 in several cancer cells, as well as in CAFs, may induce the expression of diverse genes, like c-fos, cyclin D1 and CTGF, involved in important biological effects, including cell proliferation and migration [71]. Moreover, GPER1 signalling triggers HIF-1α-dependent VEGF expression supporting its involvement in angiogenesis and progression of breast cancer [72]. Interestingly, estrogenic GPER1 activation is able to regulate the expression of the pro-inflammatory cytokine IL1β and its receptor IL1R1 in CAFs and breast cancer cells, respectively, suggesting a fine feedforward, which links tumor microenvironment with tumor cells toward the progression of breast cancer [73]. In this context, increasing data have indicated that the depletion of CAFs in tumor stroma led to decreased cancer growth and improved response to therapies [74]. These data suggest that tumor microenvironment is a fertile ground for innovative therapeutic approaches with the potential to ameliorate existing treatment and prevention options.

## 4. miRNA History and Biosynthesis

miRNAs were initially discovered in *Caenorhabditis elegans* as small temporal RNA (stRNA) [75] and characterized in subsequent studies [76,77,78]. miRNAs are a large family of small non-coding RNAs (about 22 nucleotides) presents in all species ranging from plants to humans [79]. miRNAs have regulatory and catalytic functions at the post-transcriptional level [80], by binding to the 3′- and, rarely, 5′-untranslated regions of one or more functionally related target mRNAs, thus repressing protein translation or initiating mRNA destabilization/degradation [81]. Considering that more than 60% of all mRNAs could be possible target of miRNAs [82], dysregulation of their expression may lead to important alterations in cellular processes such as cell proliferation, apoptosis, differentiation, and embryonic development [83]. Therefore, dysregulation of miRNAs expression may be associated with several pathologies including cardiovascular and blood diseases [84,85], diabetes [86] and cancer [83]. In this regard, it is important to mention that, depending on the cell context, the same miRNA may work as a tumor suppressor or show oncogenic activities [87].

**Figure 1:**
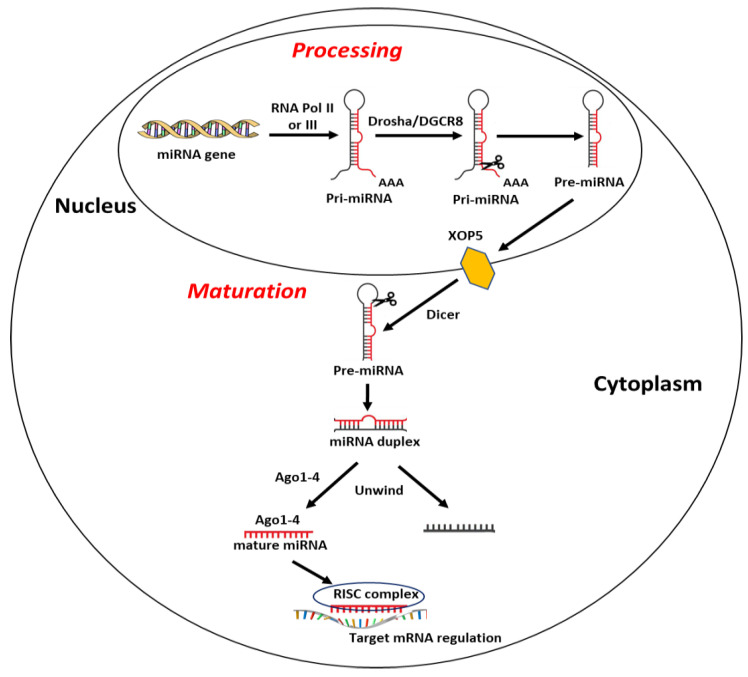
Schematic representation of miRNA synthesis, as explained in the text. Abbreviations: RNA Pol II or III: RNA Polimerase II or III; Drosha: DGCR8: DiGeorge syndrome critical region gene 8; XOP5: Exportin 5; RISC complex: RNA-induced silencing complex.

Most miRNA sequences are located within specific gene loci and about 30% of these are inside intronic gene sequences [88]. Briefly, biogenesis of miRNAs begins with their transcription in capped and polyadenylated long primary transcripts (pri-miRNAs) by RNA polymerase II or III [88]. Pri-miRNAs are then processed in the precursor miRNA (pre-miRNA), long 60–120 nucleotides, by the protein complex formed by RNase III endonuclease Drosha and the double-stranded RNA-binding protein DGCR8 (DiGeorge syndrome critical region gene 8) [89]. To prevent the nuclease degradation and easing its translocation from nucleus to cytoplasm, pre-miRNA is then assembled into the complex nucleocytoplasmic transporter factor Exportin-5 (XPO5) and Ran/GTP [90]. In the cytoplasm, the RNase II enzyme Dicer complex cuts the hairpin loop and produces a miRNA duplex of ~22 bp, containing a passenger strand, normally degraded, and a mature miRNA strand, which is loaded onto argonaute protein (Ago1–4) and united with the RNA-induced silencing complex (RISC) [91,92,93]. Finally, miRNA–RISC complex binds and regulates the expression of target mRNA [82] (Figure 1). Of note, a single miRNA may target and affect the expression of several mRNAs, and a single transcript may be targeted by numerous miRNAs [94].

## 5. miRNA Expression and Regulation in Breast Cancer

Currently, miRBase database (release 22.1) reports information about 1917 human precursors and 2656 mature miRNAs (http://www.mirbase.org/) whose ~ 2300 have been validated in a recent study [95]. The involvement of miRNAs in breast cancer development and progression it has been well demonstrated, thus miRNAs may be considered a potential diagnostic and prognostic markers, as well as therapeutic targets. To date, in November 2020, “miRNAs and breast cancer” keywords in PubMed database yielded 6227 papers, of which about 96% were published during the past decade (https://pubmed.ncbi.nlm.nih.gov/). In Table 1 are listed and referred the more representative miRNAs modulated in breast cancer [96,97,98,99,100,101,102,103,104,105,106,107,108,109,110,111,112,113,114,115,116,117,118,119,120,121,122,123,124,125,126,127,128,129,130,131,132,133,134,135,136,137,138,139,140,141,142,143,144,145,146,147,148,149,150,151,152,153,154,155,156,157,158,159,160,161,162,163,164,165,166,167,168,169,170,171,172,173,174,175], one of the first solid tumors profiled for miRNAs expression [122].

Studies performed by Iorio and colleagues revelled a miRNA differential modulation in breast cancer; for instance, the expression of miR-125b and miR-145 was found down-regulated, while miR-122 and miR-155 showed an up-regulation [122], suggesting a putative tumor suppressor or oncogenic action, respectively. miRNA-21 is the more expressed in breast tumor tissue as compared to matched normal tissue. In addition, miR-21 has been associated with advanced clinical stage, lymph node metastasis and poor prognosis [176]. Moreover, it has been reported that the over-expression of miR-21 is able to mediate cell survival and proliferation by targeting several potential tumor suppressor genes, like phosphatase and tensin homolog (PTEN) [177], tropomyosin 1 (TPM1) [178] and programmed cell death 4 (PDCD4) [179]. On the contrary, the ectopic expression of miR-205 in breast cancer cells reduced cell growth effects and improved the responsiveness to tyrosine kinase inhibitor, gefitinib [180].

miRNAs were also related to certain biological features of breast cancer, like ER and progesterone receptor (PR) expression, as well as the tumor stage and invasion [122]. In this context, it was reported that E2-ERα binding modulates miRNA-191 and miRNA-425 expression in breast cancer cells [181]. Furthermore, it has been shown that certain miRNAs may regulate ERα levels, impairing estrogenic pro-tumorogenic action in breast cancer [122]. For instance, miRNA-206 targets directly ERα [122], whereas miRNA-221 and miRNA-222 may confer a tamoxifen resistance, regulating the expression of p27 [182] and ERα [183].

**Figure 2:**
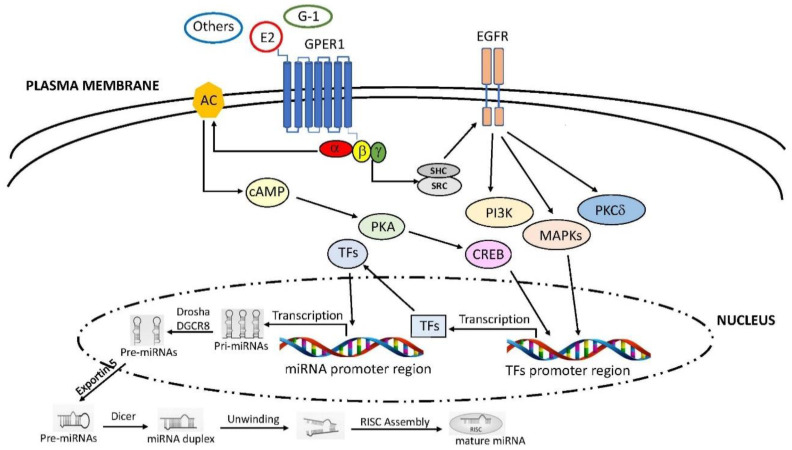
Schematic representation of GPER1 involvement in the regulation of miRNA expression. Ligand-binding to GPER1 leads to activation of SRC through the β and γ subunits of the G protein. The complex SRC tyrosine kinase-SHC adapter protein triggers the EGFR transactivation, which, in turn, stimulates downstream pathways, like PI3K, MAPKs and PKCδ. In addition, GPER activation stimulates the activity of AC through the Gα subunit, leading to the PKA-mediated increase of CREB. Both signalling may trigger the expression of specific TFs involved in miRNA transcription. In the nucleus miRNAs are processed and exported in cytoplasm, where they become mature miRNAs. SRC: steroid receptor coactivator; EGFR: epidermal growth factor receptor; PI3K: phosphatidylinositol 3-kinase; PKCδ: phospho-kinase C delta; AC: adenylyl cyclase; PKA: phospho-kinase A; CREB: cAMP response element-binding protein; TFs: transcription factors. Further abbreviations are addressed in the text.

Emerging data have reported that estrogenic stimuli are able to regulate miRNAs expression not only through the classic ERs, but also by the GPER1 involvement [184,185] (Figure 2).

In particular, it has been shown that in breast cancer cells the activation of GPER by E2 or G-1 lead to down-regulation of the tumor-suppressor miR-148a, in a dose and time dependent manner [186]. In turn, the reduced levels of miR-148a increase the expression of its target gene, named human leukocyte antigen-G (HLA-G), known to be implicated in the tumor-driven immune escape in malignancies [186]. In subsequent experiments, Tao and collaborators also reported that miR-148a downregulation mediated by E2-GPER1 signalling induces migratory effects in triple-negative breast cancer cells by overexpressing the lncRNA HOTAIR [187]. Consistent with this evidence, data obtained in ER-negative and GPER1-positive cancer types have indicated that the over-expression of miR-144, induced by E2 and G-1 through the GPER1/PI3K/Akt/ERK/Elk1 axis, lead to reduced levels of the tumor-suppressor Runt-related transcription factor-1 (Runx-1) and the consequent induction of cell cycle progression [9]. Of note, these results were also confirmed in tumor xenografts upon G-1 treatment [9]. More recently, it has been shown that in ER-negative and GPER1-positive breast cancer cells, E2 is able to modulate the expression of diverse miRNAs [8]. Among these, the tumor suppressor miR-338-3p was similar down-regulated in both cell types. In particular, miR-338-3p levels were reduced by E2 or G1 through GPER1, causing an increased cancer cell progression mediated by the augmented expression of its target gene, the proto-oncogene c-Fos [8]. Interestingly, E2 was also able to trigger a differential landscape of miRNAs in GPER1 positive and ER-negative CAFs derived from primary and metastatic breast tumors [7], suggesting a fine regulation of miRNA expression by GPER in very different cancer microenvironments. In particular, E2 modulated miRNA expression three times more in CAFs derived from the metastatic breast cancer respect to CAFs obtained from primary breast tumors. Moreover, in CAFs derived from the metastatic breast cancer, the estrogen stimulation triggered a huge miRNA increase with respect to CAFs from primary breast tumors [7], providing further findings concerning the involvement of certain miRNAs in different phases of the metastatic process [188]. Interestingly, in CAFs derived from metastatic tumor the expression of one of the most induced miRNAs, named miR-623, returned to basal levels by silencing GPER1 expression, suggesting the involvement of this receptor in the regulation of miR-623 [189].

## 6. Conclusions

Various evidence suggests that GPER plays a crucial role in breast cancer in mediating estrogenic modulation of several target genes involved in proliferation, apoptosis, migration and metastasis. In addition, studies in the past decades have helped to elucidate the importance of miRNA regulation in a number of diseases, including breast cancer. Findings that GPER activation by E2 or G1 may regulate the expression of certain miRNAs and trigger tumorigenic effects in breast cancer, provide new knowledge of the molecular mechanism involved in this neoplasia that would be useful, not only toward innovative therapeutic approaches, but also to understand the failure of treatment in oncological patients.

## Figures and Tables

**Table 1:** Main miRNAs modulated in breast cancers.

miRNA Name	Expression	Main Targets	Action	References
Let-7	Downregulated	H-Ras, HMGA2, LIN28, PEBP1	Tumor suppressor	[96,97,98,99]
miR-10b	Upregulated	HOXD10	Oncogenic miRNA	[96,100]
miR-15a and	Downregulated	Cyclin E1, E2F7	Tumor suppressor	[101,102,103]
miR-16				
miR17-5p	Downregulated	A1B1 gene, Cyclin D1, E2F1	Tumor suppressor	[96,104,105]
miR-21	Upregulated	Akt, BCL-2, BAX	Oncogenic miRNA	[101,106]
miR-22	Downregulated	KRAS	Tumor suppressor	[101,107]
miR-24	Upregulated	Oct-3/4, HIF-1α, Snail, VEGFA	Oncogenic miRNA	[101,108]
miR-26a	Downregulated	Cyclin D1, CDK1, CDK6, p21, p27, p53, MYC, RFP6/ERα/BCL6-Xl, E2F7, Cyclin E2	Tumor suppressor	[101,109,110,111]
miR-27a	Upregulated	Zinc Finger ZBTB10, MYT-1	Oncogenic miRNA	[96,112]
mir-29b	Downregulated	Akt3, VEGF, c-MYC	Tumor suppressor	[101,113]
miR-30b	Downregulated	Cyclin E2	Tumor suppressor	[101,111]
miR-30c-2–3p	Downregulated	Cyclin E1	Tumor suppressor	[101,114]
miR-31	Downregulated	WAVE3, RhoA	Tumor suppressor	[115,116]
miR-33b	Downregulated	HMGA2, SALL4, Twist 1	Tumor suppressor	[101,117]
miR-34a	Downregulated	Tumor protein D52 gene, p53, E-cadherin, N-cadherin, TGFβ	Tumor suppressor	[101,118]
miR-100	Downregulated	VEFG, mTOR/HIF-1α	Tumor suppressor	[101,119]
miR-101	Downregulated	EYA1, jagged1, Hes1, Hey1, SOX2	Tumor suppressor	[101,120,121]
miR-122	Upregulated	Pyruvate Kinase, Citrate Synthase	Tumor suppressor	[101,122]
miR-124	Downregulated	STAT3	Tumor suppressor	[101,123]
miR-124a and miR-26b	Downregulated	SerpinB2	Tumor suppressor	[101,124]
miR-125b	Downregulated	Eritropoietin receptor, ERBB2, ENPEP, casein kinase 2α, Cyclin J	Tumor suppressor	[116,122,125]
miR-126	Downregulated	VEFGA, IGFBP2, MERTK, PITPNC1	Tumor suppressor	[101,126]
miR-133a	Downregulated	LASP1	Tumor suppressor	[101,127]
miR-135b	Upregulated	LATS2, CDK2, p-YAP	Oncogenic miRNA	[101,128]
miR-138	Downregulated	E-cadherin, vimentin, N-cadherin, Snail	Tumor suppressor	[101,129]
miR-140-5p	Downregulated	Cluster of differentiation 31, MMP9, Ki-67, VEGFA,	Tumor suppressor	[101,130]
miR-143	Downregulated	ERK5, MAP3K7, Cyclin D1	Tumor suppressor	[101,131]
miR-144	Upregulated	Runx-1	Oncogenic miRNA	[9]
miR-145	Downregulated	p53-mediated repression of c-myc	Tumor suppressor	[96,122,132]
miR-146	Downregulated	NF-kB	Tumor suppressor	[96,133]
miR-148a	Downregulated	WNT-1, β-catenin, MMP7, TF4, B-cell lymphoma-2, caspase	Tumor suppressor	[134]
miR-155	Upregulated	TRF1	Oncogenic miRNA	[122,135,136]
miR-191	Upregulated	HuR, TGFβ2, SMAD3, BMP4, JUN, FOS, PTGS2, CTGF, VEGFA	Oncogenic miRNA	[101,137]
miR-191-5p	Upregulated	SOX4, caspase-3, caspase-7, p53	Oncogenic miRNA	[101,138]
miR-193a	Downregulated	WT1	Tumor suppressor	[101,139]
miR-195	Downregulated	FASN, HMGCR, ACACA, CYP27B1	Tumor suppressor	[101,140,141]
miR-200b	Upregulated	Ezrin/Radixin/Moesin (ERM)	Oncogenic miRNA	[101,142]
miR-200c and miR-141	Upregulated	SerpinB2, c-Jun, c-FosB, FOXP3, KAT2B	Oncogenic miRNA	[101,124,143]
miR-203	Upregulated	PKC-ERK, SOCS3	Oncogenic miRNA	[101,106]
miR-204	Downregulated	JAK2, BCL-2, Survivin	Tumor suppressor	[101,144]
miR-204-5p	Downregulated	PIK3CB	Tumor suppressor	[101,145]
miR-205	Downregulated	HMGB3	Tumor suppressor	[116,146,147]
miR-206	Downregulated	WBP2, p-21, CDK4, Cyclin D1	Tumor suppressor	[101,148]
miR-210	Upregulated	HIFs, GPD1L, Pax-5	Oncogenic miRNA	[101,149,150]
miR-211-5p	Downregulated	SETBP1	Tumor suppressor	[101,151]
miR-221	Upregulated	A20, PARP1	Oncogenic miRNA	[152,153]
miR-296-5p and miR-512-5p	Downregulated	hTERT	Tumor suppressor	[101,154]
miR-331	Upregulated	HER2, HOTAIR, E2F1, DOHH, PHLPP	Oncogenic miRNA	[101,141]
miR-335	Downregulated	SOX4, TNC	Tumor suppressor	[101,155,156,157,158]
miR-338-3p	Downregulated	c-FOS, ZEB2, SOX4	Tumor suppressor	[8,158,159]
miR-340	Downregulated	Rho associated coleid-coil containing protein kinase 1, CTNNB1, c-MYC	Tumor suppressor	[101,160]
miR-365	Downregulated	GALNT4	Tumor suppressor	[101,161]
miR-373/520c	Upregulated	CD44	Oncogenic miRNA	[101,162]
miR-374a	Upregulated	e-cadherin, γ-catenin, CK18, vimentin, N-cadherin, β-catenin, WIF1, PTEN, WNT5A	Oncogenic miRNA	[101,163]
miR-421	Downregulated	MTA1	Tumor suppressor	[101,164]
miR-424	Downregulated	CDK1, YAP, ERK1/2	Tumor suppressor	[101,165]
miR-455	Downregulated	CDK14, Cyclin D1, p21	Tumor suppressor	[101,166]
miR-483-3p	Downregulated	Cyclin W1, p-NPAT, CDK21	Tumor suppressor	[101,167]
miR-494p	Downregulated	PAK1, E-cadherin	Tumor suppressor	[101,168]
miR-497	Downregulated	Cyclin E1, SMAD7, CD274, VEGF, HIF-1α, Cyclin E1, E2F7	Tumor suppressor	[101,141,169,170,171]
miR-519a-3p	Upregulated	TRAIL-R2, caspase-8, caspase 7, MICA, ULBP2	Oncogenic miRNA	[101,172]
miR-543	Downregulated	ERK/MAPK	Tumor suppressor	[101,173]
miR-708	Downregulated	INF-kB subunit β, COX2, c-MYC	Tumor suppressor	[101,174]
miR-1207-5p	Upregulated	STAT2, CDKN, CDK1B	Oncogenic miRNA	[101,175]
